# Heat Shock Protein 90 (Hsp90) as a Molecular Target for the Development of Novel Drugs Against the Dermatophyte *Trichophyton rubrum*

**DOI:** 10.3389/fmicb.2015.01241

**Published:** 2015-11-10

**Authors:** Tiago R. Jacob, Nalu T. A. Peres, Maíra P. Martins, Elza A. S. Lang, Pablo R. Sanches, Antonio Rossi, Nilce M. Martinez-Rossi

**Affiliations:** ^1^Department of Genetics, Ribeirão Preto Medical School, University of São Paulo, Ribeirão PretoSão Paulo, Brazil; ^2^Department of Morphology, Federal University of SergipeAracaju, Brazil

**Keywords:** Hsp, antifungal therapy, molecular target, drug synergism, itraconazole, micafungin, 17-AAG

## Abstract

Treatment of fungal infections is difficult due to several reasons, such as side effects of drugs, emergence of resistant strains, and limited number of molecular targets for the drug compounds. In fungi, heat shock proteins (Hsps) have been implicated in several processes with the conserved molecular chaperone Hsp90 emerging as a potential target for antifungal therapy. It plays key cellular roles by eliciting molecular response to environmental changes, morphogenesis, antifungal resistance, and fungal pathogenicity. Here, we evaluated the transcription profiles of *hsp* genes of the most prevalent dermatophyte *Trichophyton rubrum* in response to different environmental challenges including nutrient availability, interaction with cells and molecules of the host tissue, and drug exposure. The results suggest that each Hsp responds to a specific stress condition and that the cohort of Hsps facilitates fungal survival under various environmental challenges. Chemical inhibition of Hsp90 resulted in increased susceptibility of the fungus to itraconazole and micafungin, and decreased its growth in human nails *in vitro*. Moreover, some *hsp* and related genes were modulated by Hsp90 at the transcriptional level. We are suggesting a role of Hsp90 in the pathogenicity and drug susceptibility of *T. rubrum* as well as the regulation of other Hsps. The synergism observed between the inhibition of Hsp90 and the effect of itraconazole or micafungin in reducing the fungal growth is of great interest as a novel and potential strategy to treat dermatophytoses.

## Introduction

Dermatophytes are pathogenic fungi and primary causative agents of superficial mycoses in humans (Brown et al., 2012). These fungi are keratinolytic and infect keratinized structures such as skin, nails, and hair in the host, giving rise to diseases (also known as dermatophytoses or tineas) such as athlete’s foot, onychomycosis, ringworm, and jock itch. Among the species belonging to this group of filamentous fungi, *Trichophyton rubrum* is the leading cause of human skin and nail mycoses and has high prevalence worldwide (Havlickova et al., 2008; Seebacher et al., 2008; Nenoff et al., 2014). Although rare, disseminated or deep dermatophytoses have been reported in immunocompromised or immunosuppressed patients (Gong et al., 2007; Marconi et al., 2010; Lanternier et al., 2013). Depending on the amplitude and the site of infection, dermatophytoses can be difficult to cure and often relapse post-treatment, even in immunocompetent individuals (Gupta and Cooper, 2008; Ghannoum and Isham, 2014).

Although a reasonable number of antifungal drugs are commercially available, majority of the clinical drugs act on the ergosterol biosynthesis pathway, thus restricting the number of cellular targets. Besides, resistance to commonly used antifungal drugs has been reported in dermatophytes and other human pathogens, rendering the choice of drug challenging and exacerbating the prospect of successful treatment (Martinez-Rossi et al., 2008; Pfaller, 2012). Therefore, novel antifungal targets have become the prime focus of several researchers in the field of medical mycology. Drug combinations and synergism have been proposed as therapeutically desirable approaches to decrease the development of resistance (Kontoyiannis and Lewis, 2002).

Evaluation of the interconnection among drug resistance, stress response, and the signaling pathways activated in these processes has been revealing key elements or the core circuitry as targets for antifungal therapy (Cowen and Steinbach, 2008; Shapiro et al., 2011). One such promising cellular candidate is the heat shock protein 90 (Hsp90) (Wirk, 2011), a molecular chaperone belonging to the highly conserved family of heat shock proteins (Hsps). These proteins rapidly accumulate in the cytosol in response to heat and environmental challenges such as antifungal drugs, oxidative stress, and heavy metal exposure among others. The heat shock response (HSR) is considered a rescue mechanism that enables the cells to cope under stressful conditions and protects from severe damage. The primary role of Hsps is to sense and assist proper protein folding and refolding, and direct them for degradation in case of misfolding, thereby assuring proteome integrity and homeostasis (Lindquist and Craig, 1988). Hsps act as molecular chaperones or transcriptional regulators in a myriad of physiological functions. These proteins are classified into several families based on their function and molecular weight, which ranges from 9 to 110 kDa. Hsps are also found in all organisms (Lindquist and Craig, 1988; De Maio et al., 2012), and are involved in the assembly of protein complexes, transport and sorting of proteins into the proper cellular compartments, cell-cycle control, and protein fate, among other functions. In fungi, Hsps have been implicated in several processes, including pathogenicity, phase transition in dimorphic fungi, and antifungal drug resistance. Hsps are synthesized as an adaptive response to stress that contributes to the survival of pathogenic microorganisms in the host (Burnie et al., 2006; Brown et al., 2010).

Heat shock protein 90 is highly abundant in cells even in non-stressful state and increases further in response to different forms of stress. However, some eukaryotes present two *hsp90* genes, one inducible and the other constitutively expressed (Taipale et al., 2010). Hsp90 can associate with several proteins involved in signaling, metabolism, cell growth, transcription, protein trafficking, chromatin remodeling, and stress response, among others (Leach et al., 2012b). It is an ATP-dependent chaperone and functions as a dimer. Each monomer presents an amino-terminal domain (NTD) that binds ATP and hydrolyzes upon association with the target proteins, a middle domain (MD) crucial for the interaction with the target proteins, and a carboxyl-terminal domain (CTD) responsible for dimerization. The energy produced by the hydrolysis of ATP is used by Hsp90 to fold the target proteins to their active conformations (Taipale et al., 2010). By chaperoning the target proteins, Hsp90 can modulate several downstream processes and regulatory cascades, thus controlling the responses to dynamic environments (Shapiro et al., 2011; Leach et al., 2012b).

Inhibitors of Hsp90 have been thoroughly searched for and some natural compounds produced by microorganisms have been isolated. These include geldanamycin, which is a benzoquinone ansamycin derived from actinomycetes, and a resorcyclic acid lactone called radicicol produced by certain fungal species (Piper and Millson, 2012). Some Hsp90 inhibitors are in clinical trial for cancer therapy and derivatives of natural compounds have been synthesized to increase efficacy and decrease side effects and toxicity (Gorska et al., 2012). In general, these inhibitors act as ATP competitors and interfere with the ATP-binding domain, which turns Hsp90 non-functional and leads to the ubiquitination and proteasome degradation of target proteins because of their aberrant conformation (Wirk, 2011). Besides their therapeutic potential, geldanamycin and its derivatives have been used to characterize the role of Hsp90 in fungal adaptation to host environment and antifungal resistance, as well as to understand their synergism with other antifungal drugs. A promising and interesting consequence of Hsp90 inhibition was that the emergence of resistance to azoles and echinocandins were reduced *in vitro* in human pathogens *Candida albicans* and *Aspergillus fumigatus*, respectively, thus validating the efficiency of these antifungal drugs in experimental infection models (Cowen, 2008; Cowen et al., 2009). Compromising the Hsp90 function was also effective against *C. albicans* and *A. fumigatus* biofilms, which are highly drug-resistant recalcitrant structures and an important cause of mortality. Targeting Hsp90 with chemical inhibitors increased the susceptibility of *C. albicans* biofilms to azoles *in vitro* and in an animal infection model, and the efficacy of azoles and echinocandins against *A. fumigatus* biofilms (Robbins et al., 2011). In *C. albicans*, inhibition of Hsp90 also affected cell wall biogenesis by disrupting the signaling pathways involved in cell wall remodeling (Leach et al., 2012a). Additionally, this also impaired the Hsf1–Hsp90 auto-regulatory circuit in *C. albicans*. The heat shock transcription factor Hsf1 governs the HSR and is a target of Hsp90. Thus, Hsp90 inhibition affected the Hsf1 regulon, consequently the regulation of HSPs and the resistance to proteotoxic stress (Leach et al., 2012a).

In this work, we have chemically inhibited Hsp90 in the dermatophyte *T. rubrum* and analyzed the effects to assess the roles played by this molecular chaperone in response to antifungal drugs, fungal pathogenicity, and regulation of other genes. To analyze drug susceptibility, three molecules with different modes of action were tested; itraconazole (ITRA), 5-Fluorocytosine (5-FC), and micafungin (MCFG) act on ergosterol biosynthesis, nucleic acids, and glucan synthesis, respectively. The antifungal effects of these drugs in synergy with the Hsp90 inhibitor against the growth of *T. rubrum* were evaluated. In order to analyze the role of Hsp90 in pathogenicity, the ability of *T. rubrum* to colonize human skin and nail in the presence of Hsp90 inhibitor was tested in an *ex vivo* model of infection. The influence of Hsp90 in the regulation of *hsp* genes and other related genes such as those encoding for the heat shock factor Hsf1 and the pH responsive regulator PacC was evaluated by transcription profile analyses in response to nutritional sources. Finally, in order to assess the adaptive response to various stress conditions, the transcriptional profile of Hsps, including Hsp90, was evaluated after exposure of *T. rubrum* to antifungal drugs and substrates present in the host.

## Materials and Methods

### *Trichophyton rubrum* Strain

*Trichophyton rubrum* strain CBS118892 (CBS-KNAW Fungal Biodiversity Centre) was cultivated in malt extract agar (MEA: 2% peptone, 2% glucose, 2% agar, pH 5.7) at 28°C. To prepare the conidia suspension, 15-day-old plates were flooded with sterile 0.9% NaCl and the suspension filtered through fiber glass to remove mycelia debris. The conidia concentration in the filtrate was estimated using a Neubauer chamber, as previously described (Persinoti et al., 2014).

### Antifungal Drug Susceptibility Test

The synergistic effect between chemical inhibition of Hsp90 and antifungal agents was tested by the following method: *T. rubrum* conidia (1 × 10^6^ per plate) were spread on the surface of MEA containing 10, 100, and 300 μM of the inhibitor 17-AAG (17-allylamino-17-demethoxygeldanamycin; InvivoGen, San Diego, CA). E-test (AB Biodisk, Solna, Sweden) gradient strips of ITRA, 5-Fluorocytosine (5-FC), or MCFG were then placed on these plates. Gradient concentration of the tested antifungal drugs ranged from 0.002 to 32 μg/mL and the results were observed after incubation at 28°C for 5 days. Plates without antifungal agents were used to assess fungal development in the presence of 17-AAG. Three independent experiments were conducted.

### *Ex vivo* Pathogenicity Test

The *ex vivo* nail and skin interaction assays were performed as described here. Autoclaved small pieces of human nail obtained from healthy donors were infected with 1 × 10^4^ *T. rubrum* conidia and incubated at 28°C for 5 days, in the absence or presence of 50, 100, or 200 μM of Hsp90 inhibitor 17-AAG. After incubation, nail fragments were observed under a light microscope to evaluate the hyphal development and fungal morphology.

Small pieces of human skin were obtained from patients who underwent abdominal surgery at the University Hospital of Ribeirão Preto Medical School, University of São Paulo, Brazil (HC-FMRP-USP). After removal of the adipose tissue, the small pieces of human skin were infected with 1 × 10^4^ *T. rubrum* conidia in the absence or presence of 200 μM 17-AAG and incubated at 28°C for 5 days. Infected skin fragments were maintained in skin graft fluid (SGF; Duek et al., 2004) supplemented with or without 200 μM 17-AAG. Scanning electron microscopy (SEM) was employed to visualize hyphal development. For this purpose, the skin fragments were fixed with 3% glutaraldehyde in 0.1% phosphate buffer (pH 7.2) at 4°C for 2 h, rinsed with 0.1% phosphate buffer (pH 7.2), and post-fixed with 1% osmium tetroxide for 2 h. Samples were dehydrated by a graded ethanol series and sputter-coated with gold to obtain a layer of approximately 200 μm thickness. The samples were viewed under a Jeol JSM -6610 LV scanning electron microscope at an acceleration voltage of 25 kV.

### Growth Conditions for Gene Expression Assays

*T. rubrum* conidia (1 × 10^6^) were inoculated in 100 mL of malt extract (ME) medium (pH 5.0) or keratin medium (KM: 2.5 g/L keratin powder, MP Biomedicals, suspended in water, pH 5.0). After shaking at 28°C for 96 h, the resultant mycelia were filtered, frozen in liquid nitrogen, and stored at -80°C for expression studies. For the Hsp90 chemical inhibition assay, fresh mycelia grown for 96 h at 28°C in ME or KM were incubated for 30 or 90 min at 28°C with 100- or 300 μM 17-AAG. For antifungal drug response assays mycelia grown in ME at 28°C for 96 h were aseptically transferred to RPMI 1640 (Life Biotechnologies—buffered with 0.167M MOPS, pH 7.0) in the absence (control) and presence of sub-inhibitory concentrations of acriflavine (ACR; 1.75 μg/mL) or terbinafine (TRB; 0.2 μg/mL), and incubated for 3 h at 28°C.

For the interaction assays, the small pieces of human skin were cleaned, infected with 1 × 10^4^ *T. rubrum* conidia, and incubated at 28°C for 96 h. The fungus was then harvested and used for total RNA extraction. For the nail interaction assay, each human nail fragment was exposed to 1 × 10^4^ *T. rubrum* conidia and incubated at 28°C for 96 h. The infected nail fragments were vortexed to release fungal mycelia for total RNA extraction and the nails discarded. The interaction assays were approved by the local Ethics Committee (Protocol No. 046/2009). Three independent experiments were conducted for each growth condition and interaction assay.

### Gene Expression Analysis

Total RNA was isolated from frozen mycelia using Illustra RNAspin Mini RNA Isolation Kit (GE Healthcare). First-strand cDNA was synthesized using the SuperScriptIII First-Strand Synthesis Super Mix for qRT-PCR kit (Invitrogen). Both RNA extraction and cDNA synthesis were performed according to the manufacturer’s recommendations. An intron flanking region of the β-tubulin gene was used as positive control to verify DNA contamination and proper cDNA synthesis, as previously described (Jacob et al., 2012).

Specific primer pairs for each *T. rubrum* gene were designed using the Primer Express v.3 software (Life Technologies) and are listed in **Table 1**. qRT-PCR reactions were carried out in a final volume of 12.5 μL, containing 6.25 μL Power SYBRGreen PCR Master Mix (Life Technologies), 1.0 μL of each primer (*hsp20*, 250 nM; *hsp60*, 500 nM; *hsp70*, 450 nM; *hsp70-like*, 350 nM; *hsp clpa*, 300 nM; *hsp88-like*, 350 nM; *hsp90*, 300 nM; *cdc37*, 400 nM; *hsp ssc1*, 400 nM; *hsp78*, 350 nM; *hsf1*, 350 nM; *pacC*, 350 nM), 2.0-μL template cDNA (50 ng), and 3.25-μL ultra-pure water. Thermal conditions for qRT-PCR were 95°C for 10 min, followed by 40 cycles of 95°C for 15 s and 60°C for 1 min. All reactions were performed in triplicate in 96-well reaction plates using the StepOnePlus Real-Time PCR System (Life Technologies). A melting curve for each gene was obtained and 2% agarose gel electrophoresis was performed to confirm the amplification of the unique product of expected size for each *hsp* gene. To determine PCR efficiency, standard curves were generated using cDNA sample at five-point, twofold dilutions and measured in triplicates. The reference genes *rpb2* and *actin* were used for data normalization as previously described (Jacob et al., 2012). Relative expression was calculated by the 2^-ΔΔCT^ method (Livak and Schmittgen, 2001). Statistical significance was evaluated by one-way ANOVA followed by the Tukey’s *ad hoc* test, using the GraphPad Prism v 5.1 Software.

**Table 1:** Primers used for qPCR assays.

Gene	Accession number^a^	Primer sequences (5’– 3’)	Amplicon length (bp)	Efficiency(%)
*hsp20*	TERG_01659	F: GCCAAGGAGGAGTTGAATCCT R: AGCCCTCTCCAATCTCGTCTT	57	95.29
*hsp60*	TERG_04141	F: AAGCGTCGTTGTCGGTAAGC R: TGTCGAAGCCACGGTTAAAGT	62	92.6
*hsp70*	TERG_01883	F: CCGCCATGAACCCTGAGA R: CGAATCGTCGTCCGATAAGAC	60	96.0
*hsp70-like*	TERG_06505	F: CACGTACTCCTGCGTGGGTAT R: TGCGGTTTCCCTGATCGT	71	96.0
*hsp clpa*	TERG_07049	F: CCGGCAGTCTCCCAAGTCT R: GTAGGCAGCAGCCATGACTTC	60	91.8
*hsp88-like*	TERG_07658	F: AAGGGTGTCACCGCTGATG R: TCAGTCTAGCCTTGAGCTTGCA	61	95.8
*hsp90*	TERG_06963	F: ACCGTGCTGCCCTTGCT R: GTGATCTCGTCGCCAGACTTG	61	96.0
*cdc37*	TERG_06398	F: GAGATCGCAACTCTAGGGTACGA R: GCCCGTCAATCCGTTTCA	64	93.5
*hsp ssc1*	TERG_03206	F: ACCGAGTCCGTCAAGAGCAT R: TCGTCGGGATTAACGGACTT	59	96.1
*hsp78*	TERG_07949	F: CCGGTCTCAGCGGTGAAA R: GGTGGGCCCAAGAAACATG	56	92.6
*hsf1*	TERG_04406	F: AGTGCTGGAGGCCGAGAAG R: TCCCGACCCGAGAGCAA	60	97.4
*pacC*	TERG_00838	F: TCCCAGCAGCCCCAAC R: ATGTGGGAGGTGATGTGGT	63	98.3

*^a^Dermatophytes genome database accession number (http://www.broadinstitute.org/annotation/genome/dermatophytecomparative).*

## Results

### Inhibition of Hsp90 by 17-AAG

**FIGURE 1:**
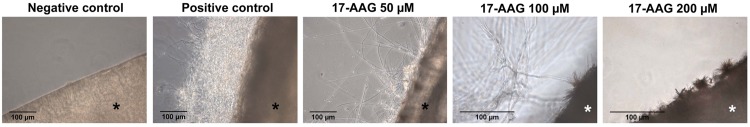
**Effect of heat shock protein (Hsp90) inhibition on *Trichophyton rubrum* growth in human nail.** Light microscopy was used to analyze hyphal development in human nail fragments infected with *T. rubrum* conidia in the absence (positive control) or presence of the Hsp90 inhibitor 17-AAG (50, 100, or 200 μM) after 5 days at 28°C. Negative control consists of uninoculated and untreated nails. Asterisks indicate nail fragments.

In order to evaluate the synergism of Hsp90 with other antifungal drugs, as well as its role in *T. rubrum* pathogenicity, 17-AAG was used to chemically inhibit Hsp90 expression in *T. rubrum*. Although inhibition of Hsp90 using 300 μM of 17-AAG had no effect on *T. rubrum* growth in MEA, an increase in fungal susceptibility to ITRA and MCFG was observed (Supplementary Figure S1). This was demonstrated by 10- and fourfold decreases in minimal inhibitory concentration (MIC) values, respectively. Moreover, there was no effect when the fungus was challenged with 5-FC (**Table 2**). The role of Hsp90 in *T. rubrum* pathogenicity was also analyzed using an *ex vivo* nail interaction assay. Inhibition of Hsp90 decreased *T. rubrum* growth on human nail *in vitro*. This decrease was dependent on the concentration of 17-AAG used, and at 200 μM, *T. rubrum* growth was almost entirely inhibited (**Figure 1**), indicating the attenuation of fungal virulence. However, SEM of *ex vivo* human skin inoculated with *T. rubrum* conidia showed no significant difference in fungal growth when Hsp90 was inhibited at the same 17-AAG concentration. (Supplementary Figure S2). This suggests that other virulence factors or incomplete inhibition of Hsp90 activity might foster fungal growth in the *ex vivo* skin model.

**Table 2:** Synergism of heat shock protein 90 (Hsp90) inhibition and antifungal drugs.

17-AAG (μM)	MIC (μg/mL)		
	5-Fluorocytosine (5-FC)	Itraconazole (ITRA)	Micafungin (MCFG)
0	>32	0.125	0.008
10	>32	0.125	0.008
100	>32	0.125	0.004
300	>32	0.012	0.002

### Expression Profile of *hsps* and Related Genes

**FIGURE 2:**
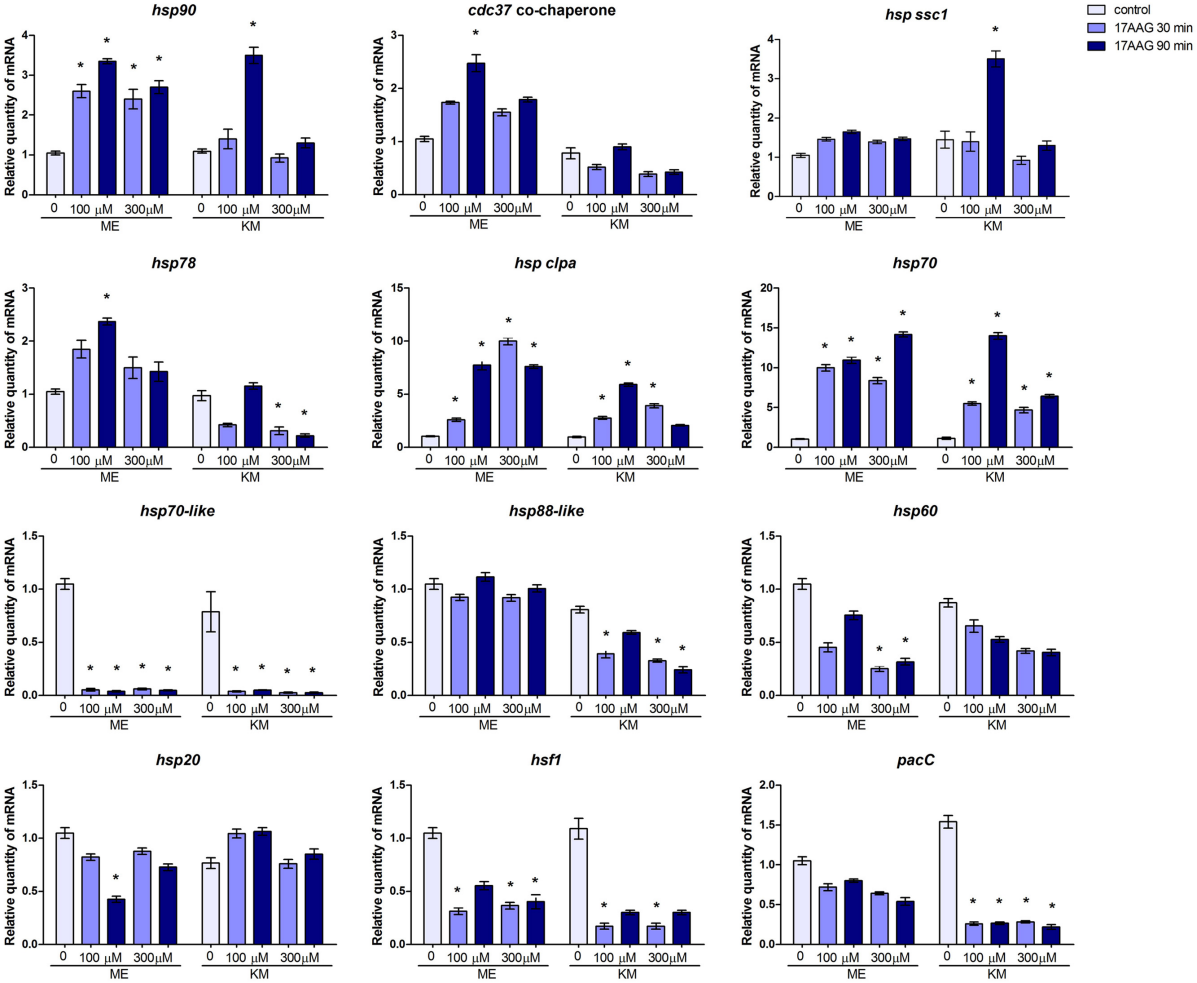
**Effect of Hsp90 on the expression of *hsp* and related genes in *T. rubrum*.** qRT-PCR analyses of the *hsp* and related genes of *T. rubrum* grown in malt extract (ME) or keratin medium (KM) in the absence or presence of Hsp90 inhibitor. Gene expression levels are represented by the quantities of mRNA in each condition relative to the control (ME). Data are represented as mean ± *SD* from three independent experiments with reactions performed in triplicate. Tukey’s *ad hoc* test was used for statistical analysis ; ^∗^*P* < 0.05.

The expression of several *hsp*s and related genes is modulated during the growth of *T. rubrum* in KM and ME in the presence of Hsp90 inhibitor 17-AAG. For some genes, this effect is time- and concentration-dependent (**Figure 2**). While the inhibition of Hsp90 led to decreased accumulation of *hsp70-like* and *hsf1* transcripts, regardless of the growth medium, the levels of *hsp60* and *hsp88-like* transcripts were decreased in response to ME and KM, respectively. However, *hsp70* and *hsp clpa* genes were upregulated in the absence of Hsp90 activity, which led to increased accumulation of *hsp90* transcripts, perhaps in an attempt to compensate for the chemical inhibition of the protein. Interestingly, *pacC* was downregulated when Hsp90 was inhibited in the presence of keratin. Moreover, the gene coding for the Hsp90 co-chaperone cdc37 was upregulated in ME but not in KM, whereas *hsp ssc1* was upregulated only in KM (**Figure 2**) upon Hsp90 inhibition. These results suggest a regulatory role for Hsp90 in the expression of *pacC* and other *hsp* genes in *T. rubrum*, depending on the growth medium.

**FIGURE 3:**
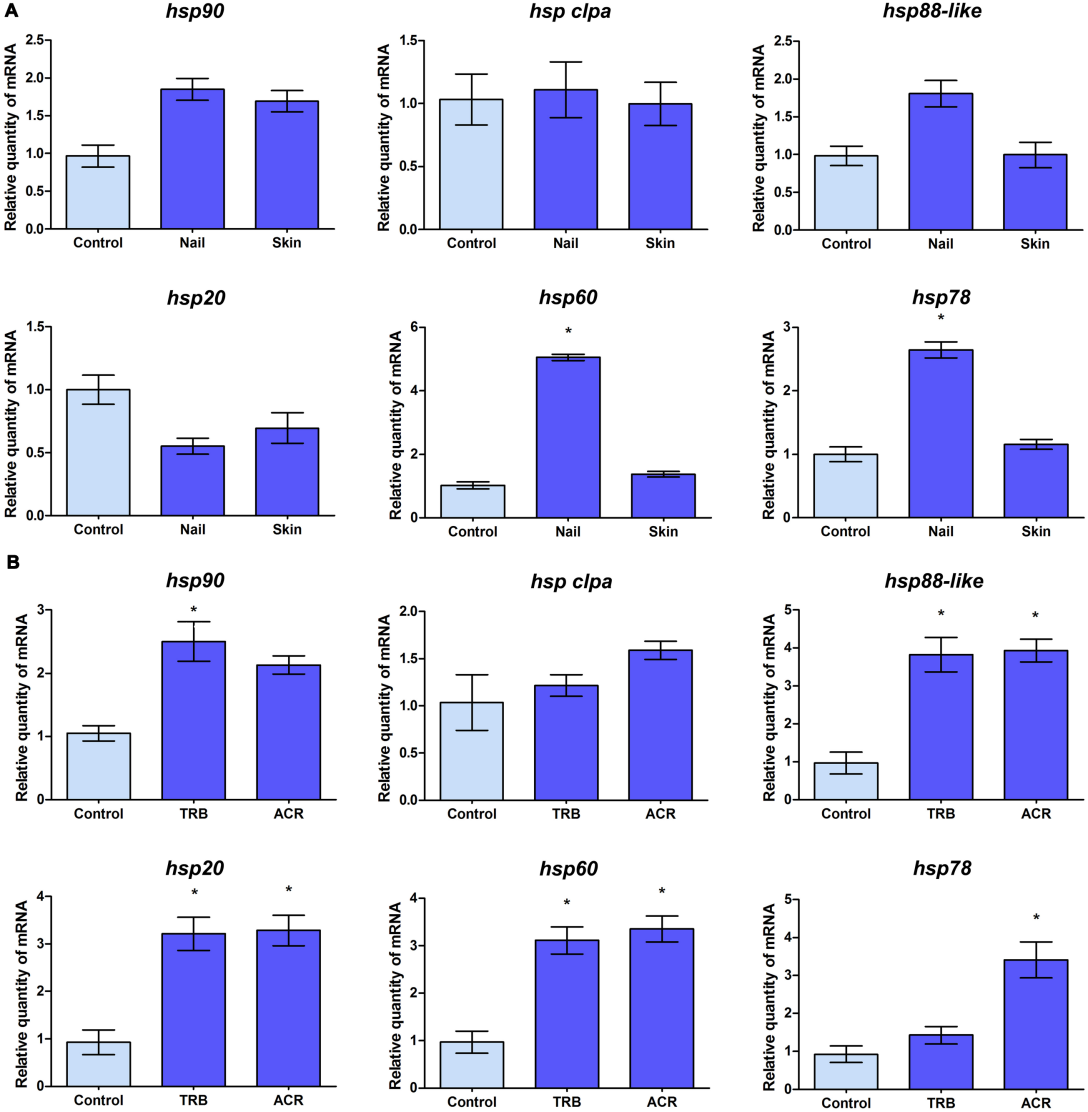
**Transcript levels of *T. rubrum hsp* genes in response to environmental challenges. (A)** qRT-PCR analyses of the transcript levels of *hsp* genes of *T. rubrum* grown in malt extract (control), human nail, or human skin fragments for 96 h. **(B)** Mycelia were transferred to RPMI medium in the absence (control) or presence of sub-inhibitory doses of terbinafine (TRB) or acriflavine (ACR) for 3 h for assessment of drug response. mRNA quantity in each condition relative to the control are represented as mean ±*SD* from three independent experiments with reactions performed in triplicate. Tukey’s *ad hoc* test was used for statistical analysis, ^∗^*P* < 0.05.

Given the involvement of Hsps in the pathogenicity of several fungal pathogens and in drug susceptibility, the expression profile of some of these *hsp* genes was evaluated in *T. rubrum* in response to other antifungal drugs, such as TRB and ACR, and during its growth on nail and skin fragments (**Figure 3**). The analysis revealed different expression profiles in response to these environmental challenges. While *hsp clpa* gene was not modulated in response to any stimuli analyzed (**Figures 3A,B**), *hsp90, hsp88-like*, and *hsp20* transcripts accumulated in response to drug exposure (**Figure 3B**). However, *hsp60* and *hsp78* presented a slightly broad range of modulation with transcripts accumulating during growth on nails and in response to one or both drugs (**Figures 3A,B**).

## Discussion

Dermatophytes affect millions of individuals annually and have become an important public health concern because of their refractivity to therapy, which prolongs the duration of treatment especially in aging populations (Coelho et al., 2008; Martinez et al., 2012). Because both the host and the pathogen are eukaryotic organisms, treatment of fungal infections is difficult due to the limited number of antifungal targets available (Martinez-Rossi et al., 2008). Another concern is the emergence of resistance to antifungal drugs currently in clinical use. Thus, it is necessary to identify new strategies for therapy against fungal infections. In this study, we observed that it is possible to increase the efficacy of antifungal drugs, ITRA and MCFG, by targeting the molecular chaperone Hsp90 with 17-AAG, an inhibitor of the Hsp90 ATPase activity. Interestingly, these antifungal agents have different mechanisms of action. While ITRA inhibits the enzyme lanosterol 14α-demethylase, thus preventing the biosynthesis of ergosterol, a key sterol in the fungal membrane (Lupetti et al., 2002; Odds et al., 2003), MCFG inhibits the enzyme 1, 3-β-D-glucan synthase, thereby blocking the biosynthesis of a key linker molecule in the fungal cell wall (Onishi et al., 2000). When challenged with antifungal drugs, several dermatophytes generally react by activating stress responses (Paião et al., 2007; Yu et al., 2007; Zhang et al., 2009; Peres et al., 2010), which often depends on the Hsp90 chaperone. Hsp90 can associate itself with a myriad of target proteins such as its co-chaperones and functional regulators, and modulate the activation and stability of the complex. Thus, functional inhibitors of the Hsp90 that act on its ATPase-coupled conformation (Siligardi et al., 2002; Lotz et al., 2003) disassemble the molecular complex of Hsp90 with co-chaperones and target proteins, thereby abrogating drug resistance and increasing the efficacy of traditional antifungal drugs (Veri and Cowen, 2014). Although Hsp90 is conserved among eukaryotes, it presents some conformational differences in fungi, especially in regions such as the ATP binding and the MDs that could be selectively targeted by the chemical inhibitors (Wider et al., 2009; Shahinas et al., 2015). Alternatively, it is possible to interfere with the Hsp90 targets or functional regulators, thus expanding the possibility to find clearer discrepancies between the pathogen and the host (Veri and Cowen, 2014). Therefore, targeting Hsp90 or other related proteins may be a viable alternative to treat fungal infections caused by *T. rubrum* and probably by other dermatophytes as well. We have also shown that the Hsp90 chaperone has a role in conferring the fungus with the ability to colonize human nails *in vitro*. The involvement of this chaperone in the pathogenicity of other pathogens such as *C. albicans* and *C. glabrata* has been demonstrated (Noble et al., 2010; Leach et al., 2012b; Singh-Babak et al., 2012). Thus, blocking the action of Hsp90 in dermatophytes becomes a potential strategy that combines this therapy with conventional antifungal drugs, which would enhance the overall outcome of the treatment.

The broad spectrum of Hsp90 functions were confirmed by changes in the expression profile of various *hsp* and related genes upon chemical inhibition of this chaperone in *T. rubrum*. Moreover, these changes in gene expression in the fungus were nutrient-dependent (ME and KM medium). The heat shock transcription factor Hsf1 positively regulates the transcription of the *hsp90* gene in *C. albicans* and *Saccharomyces cerevisiae*; both pharmacological inhibition and genetic depletion of Hsp90 correlate with Hsf1 activation in response to thermal stress (Wu, 1995; Leach et al., 2012b). However, *hsf1* transcript levels decreased when *T. rubrum* was challenged with the Hsp90 inhibitor in MEA or KM, under non-stressful temperature conditions (**Figure 2**). Additionally, there was an evident increase in *hsp90* transcripts in *T. rubrum* treated with the Hsp90 inhibitor, which was likely to compensate for the absence of Hsp90 function (**Figure 2**). This suggests a regulatory role for Hsp90 over *hsf1* transcript levels or a compensatory mechanism upon Hsp90 inhibition, in which most of the *hsf1* transcripts in the cell are efficiently transduced into protein, in turn aiding *hsp90* transcription. In *T. rubrum*, putative DNA-binding sites for Hsf1 in the *hsp90* promoter region enable direct regulation. Another interesting observation from this study is the decreased amount of the *pacC* transcripts during growth in keratin cultures containing 17-AAG (**Figure 2**). The transcription factor PacC mediates diverse metabolic events, including virulence and keratinolytic activity, in *T. rubrum* (Ferreira-Nozawa et al., 2006; Silveira et al., 2010; Martinez-Rossi et al., 2012), suggesting a correlation between the *pacC* and *hsp90* genes and fungal virulence. Additionally, we observed an increased accumulation of some *hsp* transcripts on Hsp90 inhibition, while others, including those belonging to the same family, suffered drastic decline. For example, regardless of the culture condition, transcription of the *hsp70-like* gene showed a significant reduction, whereas *hsp70* transcripts increased significantly and *hssc1* (Hsp70 family protein) transcripts practically accumulated to the same amount, suggesting different roles for each Hsp (**Figure 2**).

In order to evaluate the expression profiles of some *hsp* genes in *ex vivo* models, as well as during treatment with antifungal drugs, *T. rubrum* was cultured in MEA or in nail or skin fragments (**Figure 3A**) and in RPMI medium containing sub-inhibitory concentrations of TRB or ACR (**Figure 3B**). *hsp90* gene expression increased only in response to TRB, an antifungal used to treat dermatophytosis. Unexpectedly, *hsp90* transcript levels were similar to those observed in other experimental conditions tested, including the human nail, in which the fungus was dependent on Hsp90 function when this substrate was its sole nutrient source. It is possible that the fungus depends on similar amounts of the Hsp90 protein to cope with varying culture conditions. The transcription profile of the *hsp clpA* gene was unchanged in the analyzed conditions, demonstrating constitutive expression in response to various antifungal drugs and growth conditions. However, there was an increase in *hsp20, hsp60, hsp78*, and *hsp88-like* gene transcript levels when *T. rubrum* was challenged with ACR and/or TRB, suggesting their involvement in cellular stress responses. An increase in *hsp60* and *hsp78* transcripts was also observed when the fungus was grown in nail fragments, suggesting that Hsp60 and Hsp78 proteins act as Hsp90 co-chaperones in nail infections.

## Conclusion

Complex formation of Hsp90 and its chaperones depends on an ATPase-coupled conformational cycle, which links ATP binding and hydrolysis. This is a highly conserved mechanism in eukaryotic organisms, including *C. albicans* and other human pathogenic fungi, since ATP binding and hydrolysis are essential for Hsp90 function. Functional inhibition of ATP binding to Hsp90 disassembles the molecular complex between Hsp90 with target proteins and co-chaperones, consequently abrogating drug resistance and increasing the efficacy of traditional antifungal drugs such as ITRA and MCFG. Blocking the Hsp90 activity drastically decreased the ability of *T. rubrum* to grow on human nail fragments and interfered with the modulation of some *hsp* genes and the *pacC* gene, a regulator involved in *T. rubrum* virulence. Thus, blocking Hsp90 function in dermatophytes is suggested as a potential strategy for combination of this therapy with traditional antifungal drugs, which would enhance the antifungal efficacy. This is an attractive hypothesis to be explored further probably using *in vitro* and/or *ex vivo* models of infection as well as new inhibitors for Hsp90 and other co-chaperones.

## Author Contributions

TJ performed most of the experimental procedures, such as fungal cultivation, RNA extraction, synergism assays, and qPCR. NP participated in the experimental design and procedures, and drafted the manuscript. PS performed computational analyses to identify the genes used in this work, their promoter regions, identifying DNA-binding domains and the putative proteins. He also performed the statistical analyses of qPCR. EL performed some experimental procedures, such as skin infections, image acquisition, and drafted the manuscript. MM performed the nail infections, synergism assays and image acquisition. AR and NM-R designed the project, supervised the research study, and prepared the manuscript. All authors participated in data analysis and interpretation, read, revised critically the manuscript and approved the final version. Also, all authors agree to be accountable for all aspects of the work in ensuring that questions related to the accuracy or integrity of any part of the work are appropriately investigated and resolved.

## Conflict of Interest Statement

The authors declare that the research was conducted in the absence of any commercial or financial relationships that could be construed as a potential conflict of interest.
