# Supplementary figures and images for: Heat Shock Protein 90 (Hsp90) as a Molecular Target for the Development of Novel Drugs Against the Dermatophyte Trichophyton rubrum
Source: Front Microbiol. 2015 Nov 10;6:1241. doi: 10.3389/fmicb.2015.01241 (PMC4639609; doi:10.3389/fmicb.2015.01241)

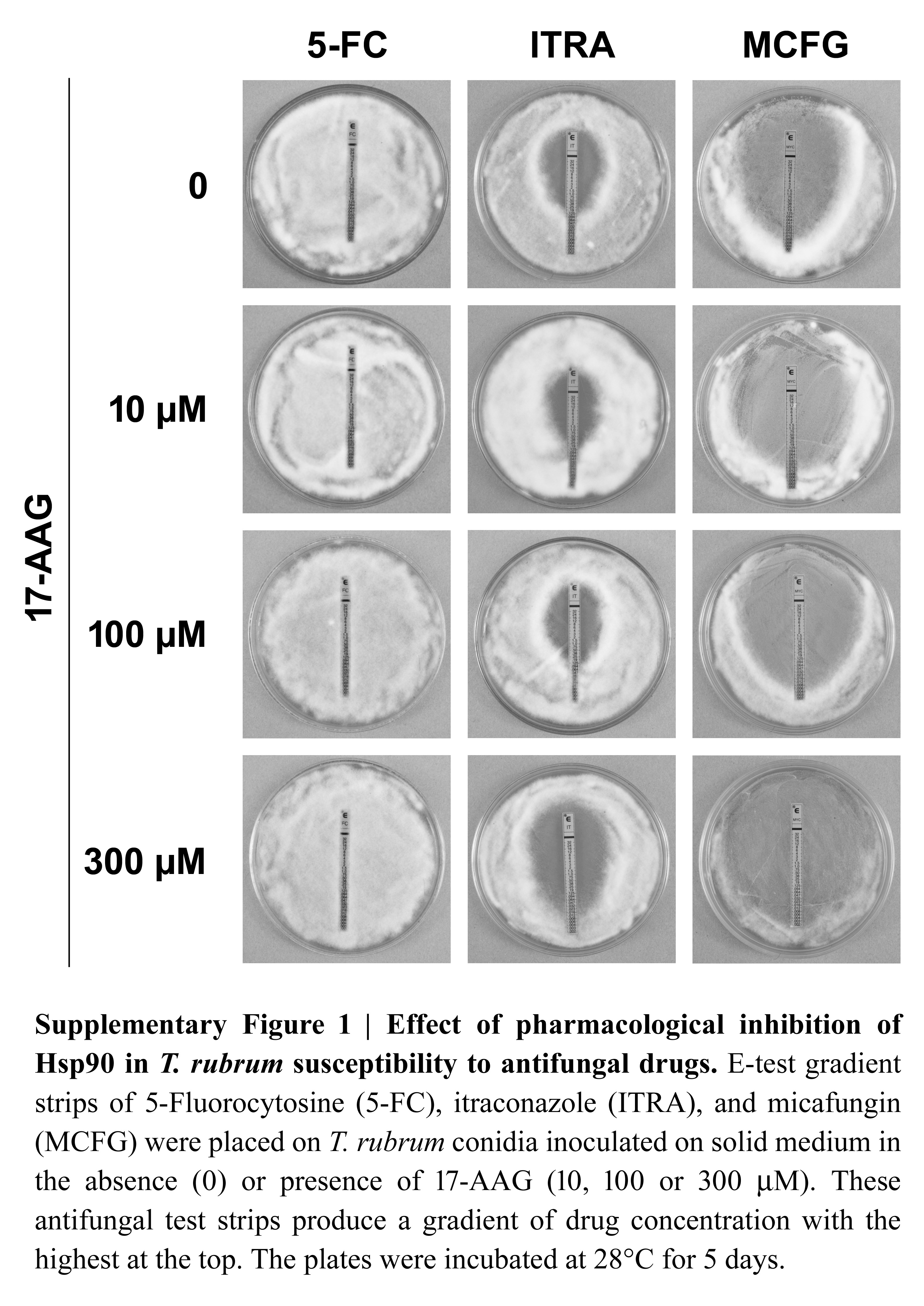

Supplement: Supplementary file 1 [file Image_1.JPEG]

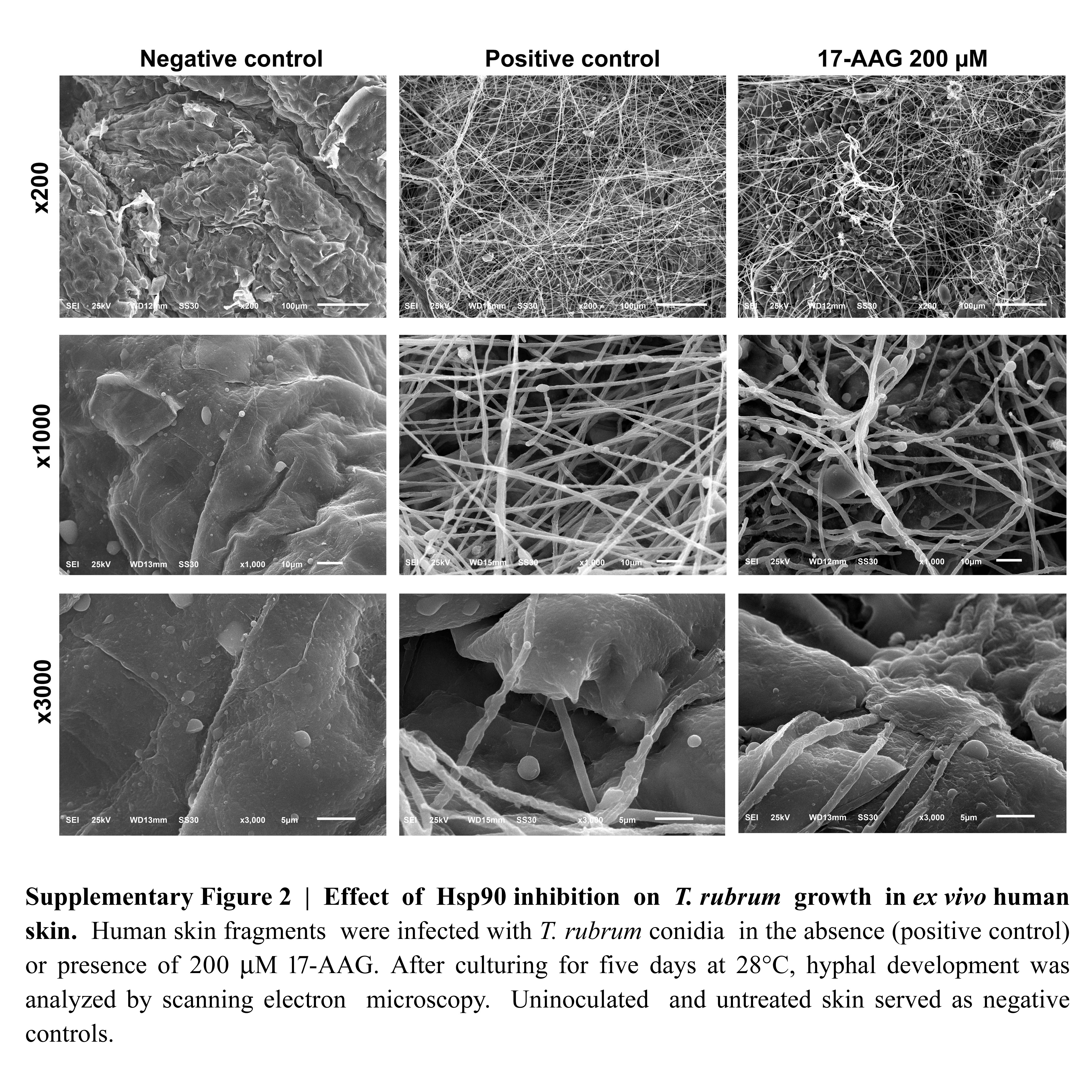

Supplement: Supplementary file 2 [file Image_2.JPEG]
